# RNA Editing and its Control in Hepatitis Delta Virus Replication

**DOI:** 10.3390/v2010131

**Published:** 2010-01-12

**Authors:** Renxiang Chen, Sarah D. Linnstaedt, John L. Casey

**Affiliations:** Department of Microbiology and Immunology, Georgetown University Medical Center, Washington, DC 20007, USA; E-Mails: rc278@georgetown.edu (R.C.); sarah.linnstaedt@gmail.com (S.D.L.)

**Keywords:** hepatitis delta virus, RNA editing, hepatitis delta antigen, ADAR1, RNA structure, RNA structural dynamics, RNA-protein interactions

## Abstract

The hepatitis delta virus genome is a small circular RNA, similar to viroids. Although HDV contains a gene, the protein produced (HDAg) is encoded by less than half the genome and possesses no RNA polymerase activity. Because of this limited coding capacity, HDV relies heavily on host functions and on structural features of the viral RNA—very much like viroids. The virus’ use of host RNA editing activity to produce two functionally distinct forms of HDAg is a particularly good example of this reliance. This review covers the mechanisms and control of RNA editing in the HDV replication cycle.

## 1. Introduction

Hepatitis delta virus (HDV) is a subviral human pathogen that increases the severity of acute and chronic hepatitis in those infected with its helper, hepatitis B virus [1]. HDV is similar to viroids in several respects: (i) the genome is a single stranded circular RNA that can form an unbranched rod structure; (ii) the RNA genome is replicated by host RNA polymerase; and (iii) replication involves a rolling circle mechanism requiring autocatalytic cleavage of the RNA by an internal ribozyme. Moreover, for both HDV and viroids, multiple RNA structural features play essential roles in the replication process.

**Figure 1.**
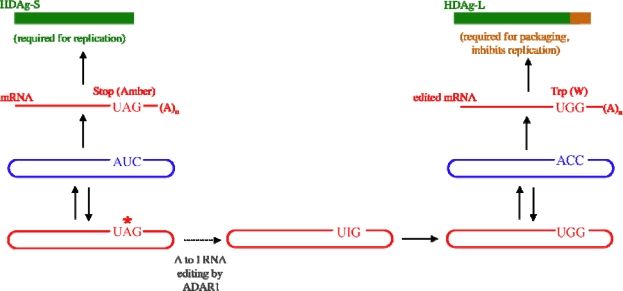
The role of RNA editing in HDV replication. Replication begins with the production of the mRNA for HDAg and the antigenome (*left*). The mRNA produced initially contains an amber stop codon and produces the 195 aa form of HDAg, HDAg-S, which is required for RNA replication. The orientation of the genome (blue) in this schematic is such that the open reading frame of HDAg, which is translated from an antigenomic sense mRNA (red), proceeds from left to right. During the course of replication, in a fraction of the antigenomic RNAs, the adenosine in the amber stop codon in the antigenome (red) is deaminated to inosine by the host RNA editing enzyme ADAR1 (bottom). Upon subsequent RNA replication, the inosine is transcribed as though it were G; as a result, the mRNA produced contains a tryptophan (W) codon instead of the amber stop codon and an additional 19–20 codons are translated to yield HDAg-L, which is required for virion packaging and inhibits replication. Because editing changes the amber stop codon to a tryptophan codon the editing site (denoted by an asterisk) is referred to as the amber/W site.

HDV differs from viroids in that the genome is larger (∼1,700 nt) and encodes a protein, hepatitis delta antigen (HDAg). HDAg serves at least two critical functions in HDV infection: participation in replication of the RNA genome, and packaging of the viral RNA with the envelope protein of hepatitis B virus. These two functions depend on two forms of HDAg, HDAg-S and HDAg-L, that differ by the presence of an additional 19–20 aa at the *C*-terminus of HDAg-L. The 195 aa short form (HDAg-S) is required for RNA replication; the 214 aa long form (HDAg-L) is not only required for RNA packaging, but also inhibits replication [2–6]. Infectious virus encodes HDAg-S, which is the only form of HDAg produced initially. HDAg-L is produced as a result of RNA editing by the host RNA adenosine deaminase ADAR [7–9]. During replication, in a limited fraction of the antigenomic RNA, this enzyme deaminates the adenosine in the amber stop codon of the HDAg gene to inosine (Figure 1).

Because inosine base pairs preferentially with C rather than U, upon subsequent replication and transcription, the amber stop codon in the hepatitis delta antigen (HDAg) open reading frame is changed to a tryptophan codon, the reading frame is extended by 19 or 20 codons and HDAg-L is produced. Thus, HDV uses the host RNA editing activity to switch the mode of replication from RNA synthesis to packaging.

From the scheme depicted in Figure 1 it is clear that editing plays a central role in the HDV replication cycle. Because HDAg-L is a limiting factor for virus production, insufficient editing reduces virus output [7,10]. Conversely, excessive or premature editing strongly diminishes viral RNA accumulation and, as a result, also decreases virus output [10–12]. Moreover, it is important to note that, unlike mRNA and miRNA substrates for RNA adenosine deamination, HDV mRNA is not edited directly. Rather, editing occurs on the antigenome, which is a replication intermediate. Thus, edited genomes and antigenomes accumulate during the course of replication. Because edited genomes are also packaged into virions, excessive editing reduces the infectivity of viral progeny. From this discussion, it is clear that optimal production of infectious virus requires control of editing.

We have found that editing is tightly controlled in three ways: it is highly specific for the particular adenosine at the amber/W site; and both the rate at which editing occurs and the fraction of RNAs edited are modulated. HDV RNA editing requires highly specific interactions between the host RNA editing enzyme, ADAR1, and structural features in the HDV RNA. Analysis of RNA editing in two HDV genotypes, types 1 and 3, has indicated that editing is essential for both, yet, the RNA structures involved and the mechanisms by which editing is controlled differ substantially. Comparison of editing in these two genotypes has revealed remarkably consistent functional requirements despite widely divergent structural characteristics, underscoring the critical role of this process, and its control, in the HDV RNA replication cycle. This review covers the mechanisms of RNA editing in the HDV replication cycle and the regulatory mechanisms by which HDV controls editing.

## 2. RNA Structural Requirements for Editing

**Figure 2.**
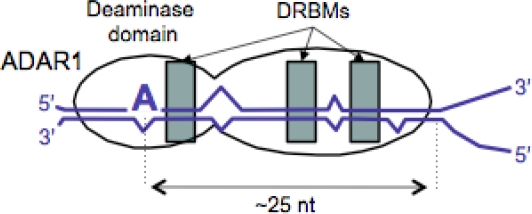
Model for interaction between ADAR1 and substrates for site-specific editing. The RNA is indicated by the blue lines; closely spaced parallel segments indicate base pairing. The target adenosine is indicated by the large blue A. Binding of ADAR1 to the RNA is mediated by the DRBMs (shaded rectangles), which recognize the dsRNA elements both 3′ of and around the targeted adenosine and position the deaminase domain.

The host enzyme ADAR1 is responsible for editing the HDV amber/W site [7,8]. No additional factors aside from HDV RNA and ADAR1 are required [9,13]. ADAR1 contains a catalytic deaminase domain along with three dsRNA binding motifs (DRBMs) [14,15]. Specific adenosines in a number of cellular mRNAs and miRNAs have been identified as substrates for editing by ADAR1 and the closely related enzyme ADAR2 [16–20]. Two general features are common to all of the sites known to be edited by ADAR1 thus far. First, none have G as the base immediately 5′ of the adenosine to be edited. In dsRNA substrates, which are subject to highly promiscuous editing, adenosines flanked by 5′ guanosines are strongly disfavored for editing [21]. Second, all exhibit considerable base pairing that surrounds the editing site and extends at least about ∼25 bp in one direction. In most cases base pairing extends 3′ of sites and includes a limited number of mismatches, bulges and small internal loops. The current model for substrate recognition by ADAR1 is that the DRBMs recognize the base-paired region in the vicinity of the site and that the bulges and internal loops serve to position the catalytic deaminase domain at the adenosine to be edited [15,19,21–24] (Figure 2). This model is based primarily on the behavior of the *Xenopus* homolog of ADAR1 on dsRNA substrates and on analysis of interactions between the closely related enzyme ADAR2 and its substrates. For example, mutational analysis has indicated that the presence of a 6 nt internal loop strongly diminished editing of a dsRNA substrate by *Xenopus* ADAR1 [22]. In the predicted secondary structure of most mammalian mRNA and miRNA ADAR1 substrates the base-paired region is contiguous with base pairing immediately surrounding the editing site, and internal loops and bulges are smaller than 6 nt. Perhaps the simplest editing site identified thus far is the ADAR2 GluR-B R/G site, which consists of a simple 28 bp stem-loop containing three single base internal loops. While the structural determinants of editing the GluR-B R/G site by ADAR2 have been analyzed extensively [24–27], the specific RNA structural requirements for editing by ADAR1 substrates have not been analyzed to the same degree.

As described below, the secondary structures required for amber/W site editing have been analyzed for two of the 8 HDV genotypes—type 1, which is the most common, and type 3, which is the most distantly related. The structures involved share the first feature common to other editing sites—the 5′ base is not G, and at least some elements of the second, the amber/W site is present in a base-paired context. However, the overall size of the structure required for type 1 and type 3 HDV editing is considerably larger than for either dsRNA substrates of *Xenopus* ADAR1 or the few substrates of ADAR2 editing that have been characterized.

### 2.1. Structural Requirements for Editing the HDV Genotype 1 amber/W Site

**Figure 3.**
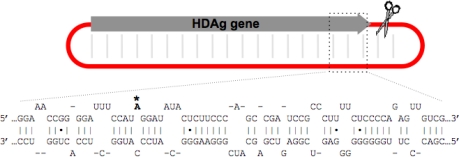
RNA secondary structure around the HDV type 1 amber/W site. The upper schematic indicates the location and orientation of the HDAg gene and, for reference, the position of the antigenomic ribozyme (scissors). A segment of the unbranched rod structure surrounding the amber/W site is shown below; the editing site is bolded and indicated by an asterisk. Site-directed mutagenesis has shown that the A-C mismatch and the eight base pairs immediately surrounding the site are necessary for editing [9,30].

The unbranched rod structure is a characteristic feature of HDV RNAs. This structure, in which about 70% of nucleotides are paired, is required for HDV RNA replication [12,28]. Site-directed mutagenesis has indicated that, for HDV genotype 1, this structure is the substrate for editing at the amber/W site [9,29]. The type 1 amber/W site occurs as an A-C mismatch pair in the midst of eight canonical Watson-Crick base pairs (Figure 3). Both the A-C mismatch and the base pairs immediately surrounding the site have been shown to be critical for editing [9,30]. A-C mismatches, which are found in some (but not all) other editing sites, have been found to maximize editing efficiency [9,30–33].

The role of base-paired regions outside the 8 bp immediately surrounding the type 1 amber/W site is not settled. Sato *et al.* concluded that no additional secondary structures were required beyond the 8bp and the A-C mismatch [34]. Inspection of the RNA secondary structure downstream of the type 1 HDV amber/W site indicates that it contains base-paired segments but is more frequently disrupted by bulges and mismatches than the region 3′ of other editing substrates (see Figure 3). These disruptions raised the question of whether the quality of base pairing in this region was sufficient to play an important role in ADAR1 binding and activity. The role of base pairing in the region 3′ of the editing site was examined by site-directed mutations that either further disrupted or increased base pairing in HDV RNA segments expressed in cells [10]. Mutations that substantially disrupted base pairing had little detectable effect on editing. Moreover, mutations that improved base-pairing, particularly in the region 15–25 nt 3′ of the editing site, increased editing significantly [10,12]. These results suggest that base pairing in the region up to 25 nt 3′ of the amber/W site is not sufficient to recruit ADAR1 to the editing site *via* interactions with the DRBMs and appear to be consistent with the conclusion of Sato *et al*. that structures outside the immediate vicinity of the amber/W site are not required for editing.

**Figure 4.**
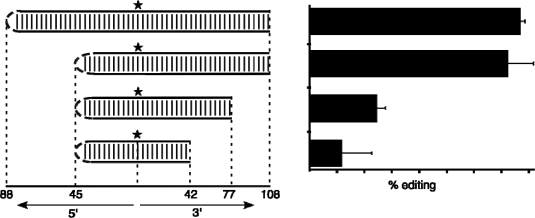
Editing at the HDV type 1 amber/W site requires sequences more than 77 nt 3′ of the site. Schematics indicate unbranched rod RNAs analyzed for editing *in vitro* by ADAR1. Numbers below indicate distance in nucleotides to the ends of the unbranched rod RNA; in the 5′ direction (left) the end is formed by a 6 nt loop, in the 3′ direction (right) the end is the 3′ end of the RNA. A 5-point star indicates the location of the amber/W site. Relative level of editing of each RNA is shown in the bar graph on the right.

In contrast to the above conclusion, more recent work in our lab suggests that sequences even further removed from the type 1 amber/W site play an important role in editing. While the previous mutational analysis indicated that base-pairing within ∼10–15 nt 3′ of the editing site might not be required for editing [10,34], analysis of RNA deletion mutants indicates that removal of sequences between 42 and 108 nt 3′ of the amber/W site reduces activity (RC, unpublished). Truncation to within 77 nt on the 3′ side of the amber/W site diminished editing to less than one-third the level of full-length RNA; further truncation to 42 nt 3′ of the site led to nearly complete loss of activity (Figure 4). Inspection of the predicted secondary structure of the region 3′ of the amber/W site indicates that the highest degree of base pairing is located from 64 to 100 nt downstream. This region includes 34 bp that are minimally disrupted by one symmetric single nucleotide internal loop and four asymmetric single nucleotide bulges. By comparison, the region 25 nt 3′ of the GluR-B R/G site, which is known to be required for editing of this site, consists of 23 bp that are disrupted by two symmetric single nucleotide internal loops [27]. To more directly determine whether the loss of editing activity in the truncated HDV RNAs shown in Figure 4 was due to the removal of these base-paired regions, we created three site directed mutations, each containing 3 nt bulges introduced into different base-paired segments in the region between 63 and 100 nt 3′ of the site. All of these mutations reduced editing to less than one-third of the wild type RNA (RC, unpublished). Based on these results, we favor a model in which the dsRNA binding domains of ADAR1 recognize the base paired region between 64 and 100 nt 3′ of the amber/W site and position the deaminase domain at the editing site *via* a long range interaction that could involve bending of the RNA.

The apparent contradiction between our recent results and those of Sato *et al.* [34] could be related to overexpression of ADAR in transfected cells in the Sato study. Several reports have indicated that such overexpression can alter the behavior of the enzyme. We have found that overexpression of ADAR1 not only increased editing at the amber/W site, but also led to high levels of promiscuous editing at other sites in the RNA [11]. Moreover, Herbert and Rich observed that, when overexpressed, a form of ADAR1 lacking the double-stranded RNA binding domains exhibited levels of activity on the GluR-B R/G site similar to that of the wild-type protein [35]. We have observed a similar result for the type 1 amber/W site—overexpression of ADAR1 constructs lacking the DRBMs edited this site with efficiency approximately half that of wild type ADAR1 (RC, unpublished). Thus, the contribution of the region 64 to 100 nt 3′ of the amber/W site to editing activity might have been missed in the Sato study because overexpression of ADAR1 decreases the requirement for the DRBMs.

### 2.2. Editing the HDV Type 3 amber/W Site Requires a Branched Structure

**Figure 5.**
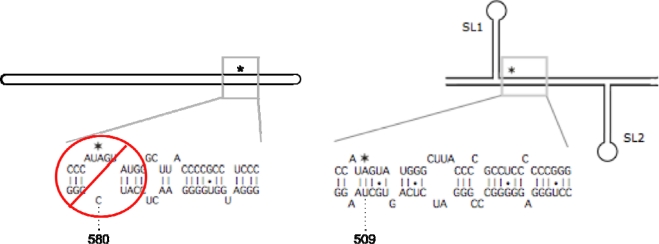
Editing at the HDV type 3 amber/W site requires a branched structure. *Left.* Schematic showing the unbranched rod structure and the RNA secondary structure in the vicinity of the target adenosine (indicated by an asterisk). This structure is not a substrate for editing by ADAR1 either in cells or *in vitro* [28,36]. *Right.* Schematic of the branched structure required for editing. The base pairing immediately surrounding the amber/W site in this branched structure is required for editing [28].

HDV type 3 also forms an unbranched rod structure that is required for replication. However, inspection of this structure indicates that the base pairing in the immediate vicinity of the amber/W adenosine is much more disrupted than in type 1 and, in fact, the type 3 amber/W adenosine is not edited when the RNA is in this conformation [28,36]. Nevertheless, this site is edited by ADAR1 during replication, just like the type 1 amber/W site [7]. What we have found is that the type 3 RNA is considerably rearranged into an alternative branched structure in order for editing to occur (Figure 5). In this structure, ca. 220 nt of the unbranched rod structure that are involved in 86 predicted base pairs are rearranged to form a branched structure consisting of two ∼25 bp stem-loops (SL1 and SL2) flanking a central base paired region that includes the amber/W site, which is itself base paired (Figure 5). Whereas the amber/W site is opposite position 580 in the unbranched rod structure, in the branched structure the paired position is 509 (Figure 5).

Because editing occurs on the antigenome, which is a replication intermediate, and replication requires the unbranched rod structure a consequence of this model is that the RNA must undergo a conformational change after editing has occurred. This conformational change is energetically favored because the branched structure required for editing is less stable than the unbranched rod structure, which is the lowest energy structure formed by the RNA [36]. Nevertheless, the branched conformation is moderately stable *in vitro*, even at 37 °C. Perhaps HDAg, or even cellular factors, play a role in facilitating the conformational change.

Analysis of the elements of the branched structure that contribute to editing activity *in vitro* has indicated that SL2, which is 3′ of the site, is essential for editing, but SL1 is required for neither editing nor ADAR1 binding (as long as the remainder of the structure is formed). Thus, the role of SL1 appears to be simply to help stabilize the structure required for editing; once the structure is formed, SL1 does not participate in the editing reaction. The requirement for SL2 is consistent with the model for ADAR1 binding to regions with double-stranded RNA character that are 3′ of the editing site. Indeed, analysis of ADAR1 binding indicates that SL2 is required. However, deletion analysis has indicated that nearly all of SL2 is required for binding (and editing); thus the linear distance along the RNA of sequence required for editing is about 40 bp—not as long as we have observed for the type 1 amber/W site (Figure 4), but considerably longer than required on dsRNA substrates [21,22] or for the gluR-B R/G site [26]. Further analysis of additional substrates of specific editing by ADAR1 may reveal whether the involvement of broader structural features, such as we have observed for the type 1 and type 3 HDV sites, is the exception or the rule.

## 3. Control of HDV RNA Editing

Control of editing at the amber/W occurs at several levels: (1) minimizing non-specific editing at sites other than the amber/W site; (2) maintaining the optimal rate of amber/W site editing to fit the timing of the viral replication cycle; and (3) preventing over-accumulation of editing.

### 3.1. Restriction of Editing to the amber/W Site

Given the important role of dsRNA secondary structure in editing by ADAR1 and the high degree of base pairing in the HDV RNA unbranched rod structure, it is clear that potential exists for editing of sites other than the amber/W site. However, such non-specific editing could be harmful to the virus. Because HDAg functions as a multimer [37], deleterious mutations of the protein have the potential to act as dominant negative inhibitors of replication. In fact, we have shown such inhibition by mutant forms of HDAg that arose as a result of spurious editing when ADAR1 or ADAR2 were overexpressed [11]. Thus, it is not surprising that promiscuous editing does not occur during typical HDV replication [11,38]; indeed, in one study we determined that, on average, the amber/W site is edited 600-fold more efficiently than the other 337 adenosines in the RNA [38].

There are three likely explanations for how HDV prevents editing from occurring at non-amber/W sites: (1) as indicated by the analysis of the type 1 amber/W site, although about 70% of bases are paired in the unbranched rod structure, this pairing is frequently disrupted by internal bulges and loops that limit ADAR binding; (2) the predicted secondary structure of type 1 RNA contains just 2 A-C mismatch pairs, which have been shown to be edited with the highest efficiency; (3) the frequency of GA dinucleotides, which are strongly disfavored for editing, is 60% higher than predicted based on a random distribution. Indeed, G is the 5′ neighbor of 48% of adenosines and both of the adenosines present as A-C mismatch pairs have G as the 5′ neighbor. In addition to the above effects that restrict the amount of editing at non-amber/W sites, HDV appears to be able to limit propagation of genomes that have been edited at these sites. We observed that editing of non-amber/W adenosines was strongly correlated with editing at the amber/W site on the same RNA [38]. Because edited RNAs encode HDAg-L, which cannot support HDV replication, such genomes are dead-end products of the virus replication cycle and virus particles containing them will not be infectious.

It is important to note that, although the amount of editing that occurs at non-amber/W sites is very low relative to the amber/W site and does not likely contribute either positively or negatively to the replication cycle, such editing does occur at low levels during replication [39] and may contribute to the evolution of genetic changes in the virus that can affect the outcome of infection [40]. Chang *et al.* were able to observe the susceptibility of additional sites to editing by using a viroid-like model in which replication of an HDAg-defective HDV genome was supported for one year by HDAg provided in *trans* [41]. Because sequence changes in this model system had no effect on HDAg and could accumulate over the course of the year, very high levels of editing were observed for a number of positions in the genome and antigenome. Thus, while in a typical analysis of editing following initiation of replication by transfecting cultured Huh7 cells, amber/W site editing reaches a maximum level of 25%–30% about 14 days posttransfection and editing at other sites is less than1% [38], in the Chang study the amber/W site was 100% edited and nine other sites were edited more than 50% [41]. It remains to be seen whether any of the additional editing sites identified in this study are preferential sites for editing during the course of HDV infection.

### 3.2. Mechanisms for Controlling Levels of amber/W Site Editing

The rate and level of amber/W site editing achieved during replication are critical because HDAg-L is the limiting factor for virus particle production, but inhibits viral RNA replication. Comparison of HDV editing with other editing substrates indicates that amber/W site editing occurs much more slowly and at lower levels. Most cellular substrates for editing are pre-mRNAs that are not only frequently edited at high levels (∼100% in some cases), but must be edited prior to splicing, which occurs within minutes following transcription. By contrast, only about 4% of HDV RNAs are edited at the amber/W site four days post transfection [7,12], and editing levels slowly increase to the maximum level. We have determined that this reduced level of editing is achieved by several mechanisms. First, for both HDV types 1 and 3, the amber/W site is sub-optimal for efficient editing. Second, both types 1 and 3 limit the availability of the RNA for editing by ADAR1, using distinctly different mechanisms. Third, because editing occurs on newly synthesized antigenome RNA, production of HDAg-L, which occurs as a result of editing and inhibits replication, eventually prevents accumulation of excessive editing levels.

### 3.3. The Type 1 and Type 3 amber/W Sites are Sub-optimal for Editing by ADAR1

**Figure 6.**
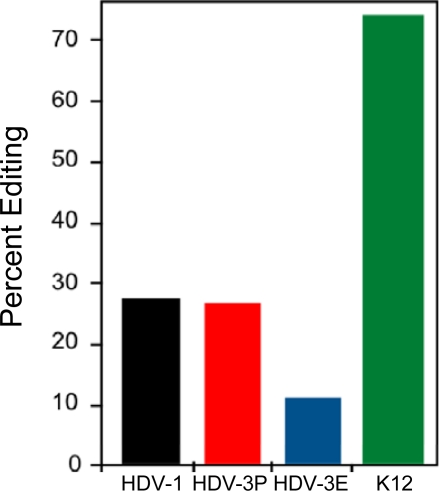
The HDV type 1 and type 3 amber/W sites are edited *in vitro* by ADAR1 less efficiently than the HHV8 K12 transcript. HDV-3P and HDV-3E are two different HDV type 3 isolates [13].

As mentioned above, previous mutational analyses had shown that increased base pairing 3′ of the type 1 editing site increased editing and substantially decreased HDV RNA replication [10,12]. These observations led to the suggestion that the secondary structure formed by HDV type 1 RNA is sub-optimal for amber/W site editing by ADAR1 [10,12]. Direct comparison of the efficiencies of ADAR1 editing of the type 1 and type 3 amber/W sites with that of another viral substrate, the HHV-8 K12 transcript [42], supports this conclusion and extends it to include the HDV type 3 amber/W site (Figure 6). The features of the sites responsible for the sub-optimal activity appear to be different for type 1 and type 3. For type 1, the lack of sufficient base pairing within ∼25 nt 3′ of the site limits activity, as described above. Increasing base pairing in this region increases both binding by ADAR1 and editing [10,43]. In contrast, base pairing is more substantial in the branched structure involved in type 3 amber/W site editing and this structure binds ADAR1 more tightly than does the type 1 unbranched rod structure (RC, SDL, unpublished). On the other hand, in the branched structure used by HDV type 3, the amber/W site exists as an A-U base pair, which is edited less efficiently than the much more common A-C mismatch [9,30–33]. This difference likely contributes to the sub-optimal nature of the site. Furthermore, we found that additional sequence and structural variations within the region ∼10 nt 3′ of the amber/W site contributed to differences in editing between the structures formed by two type 3 sequences [13] (also see Figure 6). It remains to be seen how these variations contribute to differences in editing activity. It also remains to be seen whether such variations occur among HDV type 1 amber/W sites. Finally, given the critical role of editing in the HDV replication cycle, these variations raise the obvious question of whether such differences affect pathogenesis.

### 3.4. Control of Editing by Limiting Substrate Availability—Different Mechanisms for Different Structures

In addition to limiting editing by forming sub-optimal editing sites, we have found that HDV controls editing levels by limiting the availability of this sub-optimal substrate to ADAR1. Perhaps not surprisingly, the mechanisms employed by types 1 and 3 to limit substrate availability are as different as the structures used for editing. Just as the use of different structures by these genotypes underscores the important functional role of editing in the HDV replication cycle, the use of different control mechanisms emphasizes the essential need to manage the process.

**Figure 7.**
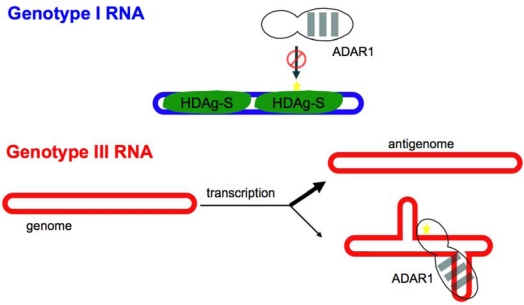
The HDV type 1 and type 3 amber/W sites are controlled by different mechanisms. Editing of this site in type 1 RNA (blue) requires the unbranched rod structure and is inhibited by HDAg (green shape), which binds this structure. Editing of the HDV type 3 RNA (red) requires a branched structure that is less stable than the unbranched rod and can therefore form only following transcription. Editing is limited by the fraction of RNA that folds into the branched conformation during transcription.

Comparison of editing occurring on non-replicating HDV type 1 RNAs with that occurring on replicating RNAs in cells indicated that editing levels were much higher for the former than the latter at early times (2–3 days) post-transfection [38]. The low level of editing occurring on the replicating RNA is consistent with the slow accumulation of replicating HDV RNA. If editing levels were too high early, replication would be inhibited by HDAg-L before sufficient HDV RNA accumulated for packaging. We and others have shown that, in transfected cells, HDAg can dramatically inhibit editing of the HDV type 1 amber/W site [12,38]. HDAg is known to bind the HDV RNA unbranched rod structure [43–45]. Recent work in our lab has shown that HDAg inhibits editing of HDV RNA by ADAR1 *in vitro*. Editing of the K12 RNA is not affected (RC, SDL, unpublished), indicating that the inhibition occurs by a direct interaction between HDAg and the RNA, rather than binding to ADAR1. Thus, our model for the down-modulation of editing during HDV type 1 replication is that the availability of the RNA for editing by ADAR1 is limited by binding of HDAg to the RNA (Figure 7). This binding likely interferes sterically with the ability of ADAR1 to bind the RNA; consistent with this interpretation, recent *in vitro* results have indicated that HDAg binding blocks degradation of the RNA by micrococcal nuclease [43]. Both HDAg-S and HDAg-L inhibit editing equally well, indicating that this mechanism for controlling editing is not directly responsive to editing levels.

The mechanism employed by HDV type 3 to control editing is quite different. For HDV type 3, editing is not inhibited by HDAg-S, either in transfected cells or *in vitro* [46]. The mechanistic explanation for this lack of inhibition is not yet clear. Possibly, HDAg does not bind the branched structure required for editing. Our recent results indicate that, for HDV type 3, editing is controlled by RNA structural dynamics - specifically, the ability of the RNA to fold into the branched structure [13]. The possibility that such a mechanism could be important was raised by the observation that the branched structure is less energetically stable than the unbranched rod structure and, therefore, can only be formed following transcription of the RNA [36]. We explored this idea using two independent HDV type 3 isolates [13]. As shown in Figure 6, *in vitro*, ADAR1 edits the amber/W site of HDV-3P RNA more effectively than that of HDV-3E RNA. However, the converse is true when editing levels attained during replication are compared. Computational analysis of the secondary structures formed by HDV-3E and HDV-3P [36] suggested that this apparent paradox might be explained by differences in the RNA structural dynamics of these two RNAs - HDV-3E RNA could form the branched structure required for editing more readily than HDV-3P RNA. Consistent with the predictions, following transcription *in vitro* HDV-3E and HDV-3P RNAs formed both the branched structure required for editing and the unbranched rod (which is not edited), but HDV-3E RNA formed the branched editing structure 3–4 fold more efficiently than did HDV-3P [13]. Based on these results, our model for down-modulation of editing of the HDV type 3 editing site is that substrate availability is limited by the fraction of the RNA that folds into the branched structure following transcription (Figure 7).

### 3.5. Negative Feedback Regulation of Editing

HDV must regulate both the rate and the extent of editing at the amber/W site because, as shown in Section 4, levels of viral RNA replication and virion production are sensitive to the kinetics and amount of HDAg-L produced. Moreover, as shown in Figure 1, editing occurs not on the mRNA, but on the antigenome, which is a replication intermediate. Hence, HDV RNA editing levels within an infected cell at any given time are the result of the accumulation of all editing events within that cell up to that time, and the percentage of antigenomes containing the UGG codon (and genomes with ACC at the corresponding positions) increases with time. The cost of this mechanism to the virus is that a fraction of viral particles contain genomes that encode HDAg-L; such genomes will not be able to replicate. Analysis of the percentage of HDV RNA edited in cells and in virus particles indicates that maximum editing levels are around 30%, and may vary for different genotypes [47,48]. Thus, it appears that HDV controls the ultimate level of editing achieved and may thus ensure continued virus viability.

Two studies have indicated that editing can be regulated by negative feedback [12,46], but the mechanisms are different. Cheng *et al.* showed that, although HDAg-S does not effectively reduce editing, HDAg-L strongly inhibits editing of a non-replicating HDV type 3 RNA [46]. These results initially led to the suggestion that regulation of editing in type 3 involves a negative feedback loop in which HDAg-L, which is produced as a result of editing, directly inhibits editing. In this event, the mechanisms for controlling ultimate editing levels would differ for HDV types 1 and 3 (for type 1 both HDAg-S and HDAg-L inhibit editing of non-replicating RNAs equally well). However, preliminary data from our laboratory indicate that this interpretation must be modified. Although the results indicated that HDAg-L alone is a potent inhibitor of type 3 editing, this protein is never present in cells replicating HDV without HDAg-S. More recent analysis has indicated that mixtures of type 3 HDAg-S and HDAg-L at ratios similar to those found in cells replicating HDV exhibit inhibitory activities similar to HDAg-S (RC, unpublished). Hence, it appears that levels of HDAg-L achieved during replication might not be sufficient to directly affect editing of HDV type 3.

We currently favor the model proposed by Sato *et al*., who suggested that HDAg-L can indirectly regulate amber/W editing by inhibiting HDV RNA replication [12]. As can be seen in the model of HDV replication (Figure 1), HDAg-L production requires HDV RNA synthesis; thus, inhibition of RNA synthesis as HDAg-L accumulates to inhibitory levels will prevent further HDAg-L production. The Sato model takes this idea one step further, and suggests that editing also requires replication of the RNA. This model is consistent with our proposed model for editing of HDV type 3 RNA - the branched structure required for editing can only be formed following RNA synthesis [13]; thus, once RNA synthesis is inhibited, no additional editing will occur. For HDV type 1, this model depends on the proposal that only newly synthesized antigenomic RNAs can be edited [12]. The mechanism in this case is not yet clearly proven, but it seems likely that it will involve the ability of HDAg to block editing at the HDV type 1 amber/W site by binding the RNA. Perhaps, only newly synthesized HDV type 1 RNA has not yet been bound by HDAg and can therefore be edited by ADAR1.

## 4. Summary and Perspectives

Editing at the HDV amber/W site expands the very limited coding capacity of this small human pathogen and plays a central role in its replication cycle. Our recent analysis of the secondary structure requirements for both the HDV type 1 and 3 amber/W sites broadens the scope of structures involved in editing to include those in which structures more than 20–25 nt away from the editing site are required. As additional cellular RNA editing substrates are identified and characterized [20], it will be interesting to see whether such extended structures are more commonly required for ADAR1 activity. Beyond the relatively large secondary structural requirements, HDV types 1 and 3 use remarkably different RNA secondary structures for editing and, consequently employ divergent mechanisms to down-modulate editing levels. For type 1, this down-modulation occurs by the common mechanism of protein sequestering the substrate, in this case by HDAg binding to the unbranched rod structure, which several studies have shown to be recognized by HDAg. The inability of HDAg to bind the branched structure required for type 3 implies that the protein is unable to bind this structure; for this HDV genotype, down-modulation of editing involves RNA folding dynamics and the potential of the RNA to adopt multiple secondary structures following transcription. Although editing levels for other editing substrates are known to vary, the regulatory mechanisms remain largely unexplored. Variations on the mechanisms used by HDV to control editing will likely play a role in many of these systems.
